# Neuropsychiatric systemic lupus erythematosus persists despite attenuation of systemic disease in MRL/lpr mice

**DOI:** 10.1186/s12974-015-0423-4

**Published:** 2015-11-06

**Authors:** Ariel D. Stock, Jing Wen, Jessica Doerner, Leal C. Herlitz, Maria Gulinello, Chaim Putterman

**Affiliations:** The Department of Microbiology and Immunology, Albert Einstein College of Medicine, Bronx, NY 10461 USA; The Department of Pathology, Cleveland Clinic, Cleveland, OH 44195 USA; Behavioral Core Facility, Department of Neuroscience, Albert Einstein College of Medicine, Bronx, NY 10461 USA; Division of Rheumatology, Albert Einstein College of Medicine, F701N, 1300 Morris Park Ave., Bronx, NY 10461 USA

**Keywords:** Lupus, SLE, Neuropsychiatric SLE, Bone marrow transplantation, Autoantibodies

## Abstract

**Background:**

Systemic lupus erythematosus (SLE) is a prototypical autoimmune disease marked by both B and T cell hyperactivity which commonly affects the joints, skin, kidneys, and brain. Neuropsychiatric disease affects about 40 % of SLE patients, most frequently manifesting as depression, memory deficits, and general cognitive decline. One important and yet unresolved question is whether neuropsychiatric SLE (NPSLE) is a complication of systemic autoimmunity or whether it is primarily driven by brain-intrinsic factors.

**Methods:**

To dissect the relative contributions of the central nervous system from those of the hematopoietic compartment, we generated bone marrow chimeras between healthy control (MRL/+) and lupus-prone MRL/*Tnfrsf6*^*lpr/lpr*^ mice (MRL/+ → MRL/lpr), as well as control chimeras. After bone marrow reconstitution, mice underwent extensive behavioral testing, analysis of brain tissue, and histological assessment.

**Results:**

Despite transfer of healthy MRL/+ bone marrow and marked attenuation of systemic disease, we found that MRL/+ → MRL/lpr mice had a behavioral phenotype consisting of depressive-like behavior and visuospatial memory deficits, comparable to MRL/lpr → MRL/lpr control transplanted mice and the behavioral profile previously established in MRL/lpr mice. Moreover, MRL/+ → MRL/lpr chimeric mice displayed increased brain RANTES expression, neurodegeneration, and cellular infiltration in the choroid plexus, as well as blood brain barrier disruption, all in the absence of significant systemic autoimmunity.

**Conclusions:**

Chimeric MRL/+ → MRL/lpr mice displayed no attenuation of the behavioral phenotype found in MRL/lpr mice, despite normalized serum autoantibodies and conserved renal function. Therefore, neuropsychiatric disease in the MRL/lpr lupus-prone strain of mice can occur absent any major contributions from systemic autoimmunity.

## Background

Systemic lupus erythematosus (SLE) is a complex autoimmune disease marked by aberrant T and B cell activation, autoantibody production, and damage in diverse end organ targets. Lupus-associated brain disease, or neuropsychiatric lupus (NPSLE), is an important driver of morbidity and mortality. Treatment of NPSLE has traditionally focused on systemic immune suppression and alleviation of symptoms, though newer biologic therapies have shown some promise [1].

Central NPLSE manifestations can be broadly categorized into two major groups: focal presentations resulting predominately from cerebrovascular disease and diffuse disorders consisting of depression, memory loss, and cognitive decline. The pathogenic mechanism underlying focal NPSLE is often a coagulopathy present in SLE patients [2–4]. Diffuse NPSLE, however, remains poorly understood.

Systemic humoral factors may be responsible for diffuse NPSLE presentations. These include anti-*N*-methyl-d-aspartate receptor (NMDAR) antibodies [5], anti-ribosomal-P antibodies [6], and complement-mediated cytotoxicity [7]. However, the mechanisms through which these humoral effectors may traverse the blood brain barrier (BBB) are not known [8]. Additionally, in both human lupus and murine models, neuropsychiatric disease can develop prior to overt systemic disease manifestations [9, 10], suggesting a possible role of brain-derived effectors in the development of NPSLE.

One spontaneous murine model, the MRL/*Tnfrsf6*^*lpr/lpr*^ (MRL/lpr) strain, is exceptionally valuable in the study of many features of SLE [11]. MRL/lpr mice develop an overall clinical and immunologic phenotype with many similarities to human lupus, including elevated titers of autoantibodies, skin and renal disease, depression-like behavior, and learning/memory deficits [12]. Moreover, the MRL/lpr mouse has a congenic control, the MRL/*Tnfrsf6*^+/+^ (MRL/+) mouse, which does not display significant autoimmune manifestations or neuropsychiatric disease until a much older age (median lifespans 17 and 73 weeks, respectively). Various contributors to the pathogenesis of NPSLE in MRL/lpr mice have been proposed, including autoantibodies to the central nervous system (CNS) antigenic determinants, abnormal cytokine expression systemically and intrathecally [13], and the development of a cellular infiltrative process targeting the circumventricular organs [14]. It is unclear whether NPSLE behavioral manifestations in either MRL/lpr mice or humans develop as a secondary consequence of systemic disease processes or if there are additional driving or inciting factors intrinsic to the CNS. This limited understanding has confounded clinicians’ ability to effectively treat this particularly debilitating consequence of SLE. In the present study, our aim was to shed light on this question through the use of bone marrow (BM) chimeras. By generating chimeric MRL/lpr mice reconstituted with healthy bone marrow, we sought to determine the relative contributions to NPSLE that are CNS driven, outside the context of systemic autoimmunity.

## Methods

### Animals

Female MRL/lpr (stock #485) and MRL/+ (stock #486) mice at 6–8 weeks of age were purchased from Jackson Laboratories (Bar Harbor, ME) and housed at 21–23 °C on a 12:12-h light to dark cycle. All animal protocols were approved by the institutional animal care and use committee.

Bone marrow transplantation (BMT) was performed essentially as described elsewhere [15]. Eight to nine week old mice were given a lethal dose of γ-irradiation (two doses of 5.5 Gy, 4 h apart). BM cells isolated from MRL/+ or MRL/lpr mice were then immediately transferred (3–5 × 10^6^ cells/mouse) via tail vein injection as follows: MRL/lpr → MRL/lpr (the first strain denotes the donor, and the second strain is the recipient), MRL/+ → MRL/+, and MRL/+ → MRL/lpr. The MRL/lpr → MRL/+ chimera was not studied due to the wasting syndrome associated with this donor-recipient pair, described extensively elsewhere [15]. Importantly, MRL/lpr → MRL/+ chimeric mice die prematurely due to a mechanism distinct from wild type MRL/lpr mice [16].

Apart from monitoring body weight, mice were left to recover for 4 weeks after transplantation. Engraftment was monitored by genotyping peripheral blood for the *Fas* gene variant. To provide a reference for typical disease manifestations, unmanipulated MRL/lpr and MRL/+ mice were included as positive (diseased) and negative (healthy) controls, matched according to age post-transplantation in the chimeric groups. Two mice in the MRL/+ → MRL/+ group and one mouse in the MRL/+ → MRL/lpr group were sacrificed 14 days post-transplantation due to wasting, indicative of bone marrow engraftment failure. All remaining transplanted mice reached the study endpoint, presumably owing to the effect of irradiation in hindering immune activity. For serum analyses and behavioral studies, the number of mice in each group was as follows: MRL/lpr → MRL/lpr, *n* = 13; MRL/+ → MRL/+, *n* = 12; MRL/+ → MRL/lpr, *n* = 18; MRL/+, *n* = 5; and MRL/lpr, *n* = 5. For other analyses including renal histology, immunofluorescence, Fluoro Jade C staining (qualitative), and PCR, a randomly selected subset was studied: MRL/lpr → MRL/lpr, *n* = 7; MRL/+ → MRL/+, *n* = 7; MRL/+ → MRL/lpr, *n* = 11; MRL/+, *n* = 5; and MRL/lpr, *n* = 5. For quantitative analysis of Fluoro Jade C staining, a random subset of stained samples was analyzed: MRL/lpr → MRL/lpr, *n* = 3; MRL/+ → MRL/+, *n* = 3; MRL/+ → MRL/lpr, *n* = 4.

### Assessment of systemic disease

MRL/lpr mice spontaneously develop hypergammaglobulinemia, autoantibodies directed against nuclear antigens, and renal disease [16]. Serum IgG and IgG anti-double stranded (ds) DNA antibody levels were measured at the time of sacrifice (27 weeks of age), as previously described [17]. Blood was not sampled prior to bone marrow transplantation to limit the possibility of post-procedural infectious complications. Urinary albumin (Bethyl Labs, Montgomery, TX) and creatinine (BioAssay Systems, Hayward, CA), as well as serum blood urea nitrogen (BUN) (BioAssay Systems), were determined at the time of sacrifice. Renal histopathology was analyzed as described previously [18], by a nephropathologist blinded to the group assignments.

### Behavioral assessment

A panel of behavioral tests extensively validated in MRL/lpr and MRL/+ mice was used for phenotypic characterization, as described previously in detail [10, 12, 19]. These tests included forced swim (FS) to assess depression-like behavior, object placement (OP), and object recognition (OR) tests to assess learning/memory, as well as open field (OF) testing to examine locomotion and exploration. The tests were used to evaluate whether the behavioral phenotype of MRL/lpr mice would manifest absent systemic autoimmunity. Chimeric mice underwent behavioral testing between 24–26 weeks of age, corresponding to 16–18 weeks post-transplantation. Before each test, mice were exposed to the testing room under low incandescent light for at least 30 min. All tests were recorded using Viewer tracking software (mid-point detection, Bioobserve, Bonn, Germany). Manually scored tests (FS, OP, and OR) were validated by a blinded observer.

### RANTES Real time quantitative PCR

RNA isolation and real time PCR (in triplicate) for RANTES was performed as described elsewhere [12]. The primers used for amplification were forward 5′-GCAAGTGCTCCAATCTTGC-3′, reverse 5′-CTTCTTCTCTGGTTGGCAC-3′. Reported fold changes of gene expression are relative to unmanipulated MRL/+ mice.

### Tissue preparation and immunofluorescence

At 27 weeks of age, mice were transcardially perfused with ice-cold PBS followed by immediate brain isolation. A portion of the brain including cortex and hippocampus was dissected and snap frozen in liquid nitrogen for subsequent RNA isolation. The remainder was dissected along the mid-sagittal plane and fixed in 4 % paraformaldehyde/PBS for 36–48 h at 4 °C. Brains were then either cryo-protected in 30 % sucrose/PBS at 4 °C for frozen sectioning or paraffin-embedded.

All immunofluorescent staining was done after blocking in 20 % normal horse serum. Representative images of IBA-1 staining were taken using a Leica SP2-AOBS confocal microscope, while RANTES and fibronectin staining were visualized using a Zeiss Axio Observer CLEM instrument. Evaluation of IBA-1 (three sections per mouse at 40–60 μM intervals, rabbit anti-IBA-1, Wako, Osaka, Japan), RANTES (goat anti-RANTES, Santa Cruz Biotechnology, Dallas, TX), NeuN (mouse anti-NeuN, Millipore, Darmstadt, Germany), IgG (Donkey anti-IgG, Jackson Immunoresearch Laboratories, West Grove PA), and fibronectin (rabbit anti-fibronectin, Abcam, Cambridge, MA) were performed on 5-μM paraffin sections. Tissue deposition of fibronectin was analyzed by subtracting the vascular fluorescent area from the total tissue fluorescent area. IBA1+ cellular infiltration was determined as either the presence of clustered IBA1+ cells indicating infiltration from the periphery, compared to isolated IBA1+ cells, indicative of resident cells within the choroid plexus. IgG deposition was calculated as the mean fluorescent intensity in the hippocampal and cortical regions of interest. All secondary antibodies were from Jackson Immunoresearch Laboratories (West Grove, PA). Following immunostaining, all sections were counterstained with DAPI, and the images analyzed using List source of imageJ (U. S. National Institutes of Health, Bethesda, Maryland, USA). Primary and secondary antibodies were withheld from several sections to control for background fluorescence and non-specific staining.

### Fluoro Jade C staining

Ten to twelve micron frozen sections were used for Fluoro Jade C (FJC) staining. After warming to room temperature, slides were immersed for 1 min each in 100 and 70 % ethanol, followed by rinsing with water. Slides were blocked for 15 min in 0.06 % KMnO_4_, rinsed, and stained in a 0.001 % solution of FJC (Millipore, Darmstadt, Germany) in 0.1 % acetic acid for 30 min. Slides were then washed, dried at 60 °C, cleared in xylene, and mounted in DPX mounting medium (Sigma, St. Louis, MO). The number of FJC+ cells in representative samples from each group, consisting of three high-powered fields from randomly selected MRL/+ → MRL/+ (*n* = 3), MRL/lpr → MRL/lpr (*n* = 3), and MRL/+ → MRL/lpr (*n* = 4) mice, were manually counted.

### Statistics

All statistical analysis was performed using GraphPad Prism software (La Jolla, CA). Normality was determined with the Kolmogorov-Smirnov test and, for most experiments, significant effects between groups of mice were determined by one-way ANOVA, followed by post hoc analysis by Fisher’s least significant difference test. Unmanipulated MRL/+ and MRL/lpr mice were evaluated using a one-tailed Student’s *t* test. For OP and OR tasks, the threshold for preference was set at >53 %. “Preference” vs. “no preference” was then analyzed by chi-square and Fisher’s exact test between groups, as described elsewhere [20]. Analysis of RANTES RT-qPCR was done with the Kruskal-Wallis test of variance followed by post hoc analysis by Dunn’s multiple comparison test. Data is displayed as the mean ± SEM, except for OP and OR, which are displayed as the number of mice in each category. For all analyses, significance was defined as *p* < 0.05.

## Results

### Transplantation of MRL/+ bone marrow to MRL/lpr mice minimizes extra-cranial disease manifestations

**Fig. 1:**
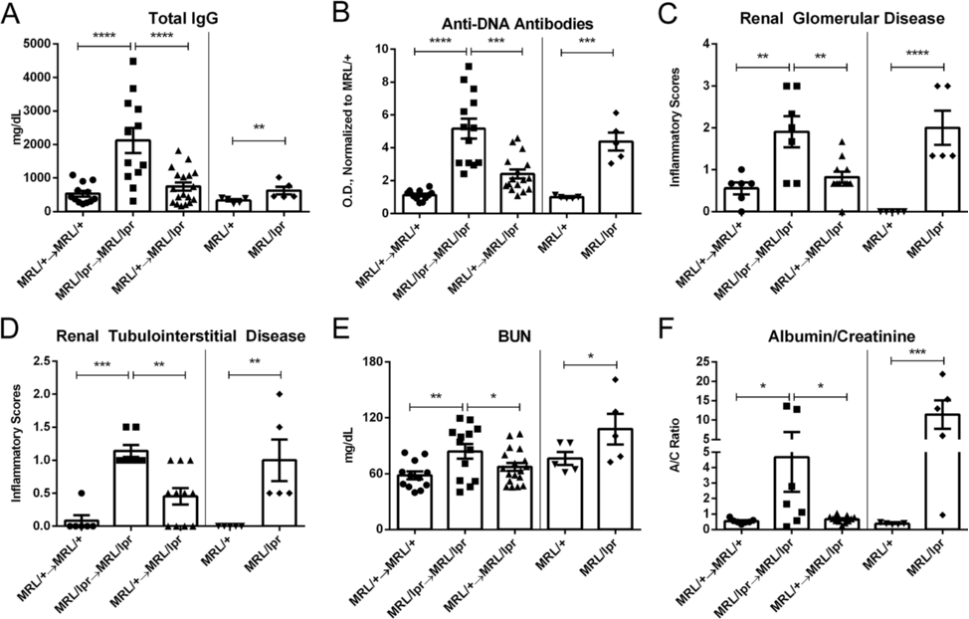
MRL/+ → MRL/lpr mice are rescued from systemic disease. Total IgG was reduced in MRL/+ → MRL/lpr mice to levels similar to MRL/+ → MRL/+ mice (**a**), as were anti-DNA antibodies (**b**) and renal histopathological scores (**c**, **d**). MRL/+ → MRL/lpr mice had improved renal function, demonstrated by BUN and albumin/creatinine ratios (**e**, **f**). Data in (**b**) is expressed as mean values, normalized to unmanipulated MRL/+ mice. Measurements in this figure were performed on serum obtained at sacrifice (27 weeks of age). *Error bars* = SEM, *p < 0.05, **p < 0.01, ***p < 0.001, ****p < 0.0001

MRL/lpr mice spontaneously develop hypergammaglobulinemia, anti-dsDNA antibodies, and immune-mediated glomerulonephritis. In contrast, MRL/+ → MRL/lpr chimeras displayed significantly attenuated systemic disease at 26 weeks of age. Although total IgG (F_2,39_ = 15.09; *p* < 0.0001, Fig. 1a) and anti-dsDNA antibody levels (F_2,38_ = 26.4, *p* < 0.0001, Fig. 1b) were significantly elevated in MRL/lpr → MRL/lpr chimeric mice, those of MRL/+ → MRL/lpr mice were comparable to MRL/+ → MRL/+ mice. Histologically, the kidneys of MRL/+ → MRL/lpr mice were devoid of significant glomerular (F_2,21_ = 9.211, *p* = 0.001, Fig. 1c) or tubulointerstitial inflammation (F_2,21_ = 14.34, *p* = 0.001, Fig. 1d). Renal function in MRL/+ → MRL/lpr chimeras was preserved as well, as indicated by BUN levels (F_2,40_ = 4.408, *p* = 0.01, Fig. 1e) and urinary albumin/creatinine ratios (F_2,22_ = 4.730, *p* = 0.2, Fig. 1f). Antibody titers and the assessment of kidney function and histopathology are summarized in Table 1. Collectively, these experiments confirm that BMT from MRL/+ to MRL/lpr mice markedly diminishes generalized systemic autoimmunity and provides protection from nephritis [15]. These results strongly suggest, therefore, that any development and/or persistence of an NPSLE phenotype in MRL/+ → MRL/lpr chimeric mice would less likely be attributable to systemic immune abnormalities.

**Table 1:** Antibody titers and renal function of chimeric and unmanipulated mice

Table 1	Total IgG (mg/dL)	Anti-DNA antibodies (O.D./MRL/+)	BUN (mg/dL)	Urinary albumin/creatinine (ratio)	Total renal inflammatory score	Renal deposits	Renal endocapillary proliferation	Renal crescents	Renal tabular casts	Renal interstitial inflammation
MRL/+ → MRL/+	0.99***	1.13***	0.76**	1.41	0.37***	0.67***	1.00**	0.00	0.17*	0.00***
MRL/lpr → MRL/lpr	4.56	4.17	1.10	12.20	1.60	2.57	2.71	0.44	1.00	1.29
MRL/+ → MRL/lpr	1.32**	2.40***	0.88	1.72	0.67**	1.09**	1.37*	0.00	0.45	0.45**
MRL/+	1.00	1.00	1.00	1.00	0.00	0.00	0.00	0.00	0.00	0.00
MRL/lpr	1.86^##^	4.38^###^	1.41	29.84^###^	1.60^###^	2.80^###^	2.80^###^	0.40	0.60	1.40^###^

Histopathology is scored as a range of 0–5

*p < 0.05; **p < 0.01; ***p < 0.001, compared to MRL/lpr → MRL/lpr; ^#^ *p* < 0.05; ^##^ *p* < 0.01; ^###^ *p* < 0.001, compared to MRL/+

### The MRL/lpr NPSLE phenotype occurs independently of systemic autoimmunity

#### Open field test

**Fig. 2:**
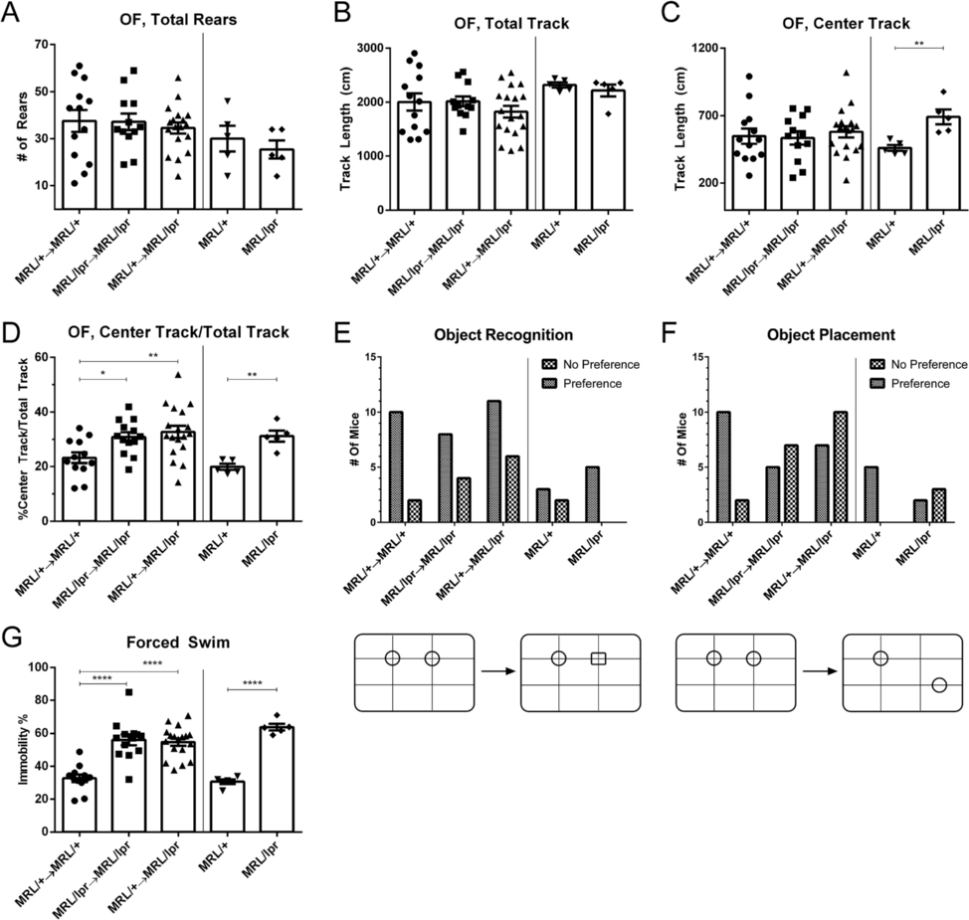
Chimeric MRL/+ → MRL/lpr mice display a behavioral phenotype consistent with MRL/lpr → MRL/lpr mice. All transplanted murine strains displayed an equivalent exploratory drive (**a**) and locomotion (**b**, **c**). An increased ratio of center track/total track, an indicator of increased risk seeking behavior, was found in MRL/+ → MRL/lpr, MRL/lpr → MRL/lpr, and MRL/lpr mice (**d**). While there were no significant differences between mice on object recognition tasks (**e**), MRL/+ → MRL/lpr (*p* < 0.05) and MRL/lpr → MRL/lpr (*p* < 0.05) displayed significantly poorer performance on spatial memory tasks as compared to MRL/+ → MRL/+ mice (**f**). The forced swim test demonstrated marked depression-like behavior in MRL/+ → MRL/lpr mice, similar to that seen in MRL/lpr → MRL/lpr and MRL/lpr mice (**g**). Immobility was not seen in MRL/+ → MRL/+ and MRL/+ mice. *Error bars* = SEM, *p < 0.05, **p < 0.01, ****p < 0.0001

Open field testing was done to evaluate overall exploratory activity, as well as to insure that BMT caused no deleterious effects on locomotion. There were no significant differences in the number of rears between groups (F_2,40_ = 0.25; ns, Fig. 2a), suggesting similar levels of exploration. Similarly, no significant difference was found in total track lengths between individual groups (F_2,40_ = 0.875; *p* = 0.03, Fig. 2b), indicating no motor deficits or musculoskeletal disability. Although center track length was similar between the groups (F_2,40_ = 0.2445; ns, Fig. 2c), there was a significant difference between unmanipulated MRL/lpr and MRL/+ mice when analyzed independently. Furthermore, both MRL/lpr → MRL/lpr and MRL/+ → MRL/lpr mice had significantly greater center-to-total track length ratios. Given the natural tendency of mice to display thigmotactic behavior, increased relative exploration of the exposed (center) portion of an open field arena is an aberrant exploratory phenotype, consistent with increased risk-taking [21, 22] (F_2,40_ = 5.382, *p* < 0.01, Fig. 2d). These effects are not a consequence of the transplantation but rather are specific neurobehavioral deficits, as the syngeneic chimeric mice did not differ from the matched unmanipulated control MRL/lpr and MRL/+ background strains.

#### Object recognition and object placement tests

The object recognition (OR) and object placement (OP) tests measure recognition and visuospatial memory, respectively [23]. Mice have a natural tendency to preferentially explore novel stimuli, including a novel object in an arena (OR) or an object that is placed in a novel position within an arena (OP) [24], as illustrated in Fig. 2e, f. Consistent with previous studies [12], there were no differences in OR performance between MRL/+ and MRL/lpr background mice (chi-square test, *χ*^2^ = 3.72, *df* = 4, ns, Fig. 2e), regardless of transplant condition. However, both MRL/lpr → MRL/lpr (*p* = 0.04) and MRL/+ → MRL/lpr (*p* = 0.03) mice displayed significantly defective visuospatial memory as compared to MRL/+ → MRL/+ mice (chi-square test, *χ*^2^ = 10.64, *df* = 4, *p* = 0.03, Fig. 2f).

#### Forced swim test

When placed in water, mice have a natural tendency to struggle or swim, whereas increased immobility is indicative of behavioral despair or depression-like behavior [25, 26]. Depression-like behavior in MRL/lpr mice has been validated by multiple metrics; forced swim immobility is the most robustly reproduced [10, 12, 27], with MRL/lpr mice exhibiting higher levels of immobility than MRL/+. In the chimeric strains, MRL/+ → MRL/lpr mice had no attenuation of this phenotype, with profound immobility equivalent to MRL/lpr → MRL/lpr and unmanipulated MRL/lpr mice (F_2,40_ = 22.67; *p* < 0.0001, Fig. 2g). In contrast, MRL/+ → MRL/+ mice had levels of immobility similar to unmanipulated MRL/+ mice.

In summary of the behavioral assessments, MRL/+ → MRL/lpr mice displayed a neuropsychiatric phenotype surprisingly similar to MRL/lpr → MRL/lpr mice, despite overall rescue from systemic disease. The presence of depression-like behavior and spatial memory deficits were consistent with unmanipulated control MRL/lpr mice, while MRL/+ → MRL/+ controls had no such behavioral deficits. Considering the prominent role autoantibodies are believed to play in the pathogenesis of NPSLE [28, 29], the persistence of the neurobehavioral lupus phenotype in MRL/+ → MRL/lpr chimeric mice despite near-resolution of systemic autoimmunity was quite unexpected.

#### The blood brain barrier (BBB) is breached in MRL/+ → MRL/lpr chimeras

**Fig. 3:**
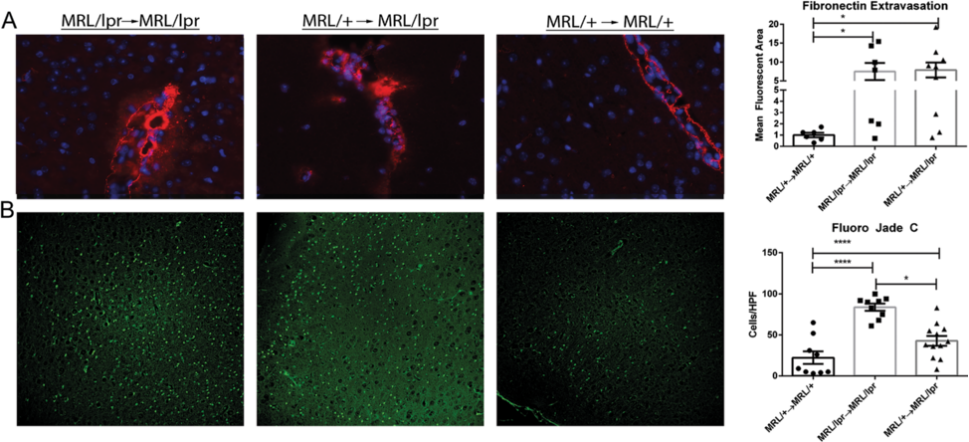
Brains of MRL/+ → MRL/lpr mice are phenotypically similar to MRL/lpr → MRL/lpr mice. Staining for fibronectin (**a**, in *red*), indicative of BBB disruption, demonstrates extravasation of this serum protein into the brain tissue of MRL/+ → MRL/lpr and MRL/lpr → MRL/lpr chimeric mice, while unmanipulated MRL/+ (not shown) and MLR/+ → MRL/+ mice had staining restricted to the vascular lumen. Fluoro Jade C staining (**b**, in *green*) revealed increased degeneration in the cortices of MRL/+ → MRL/lpr and MRL/lpr → MRL/lpr mice. Representative images of fibronectin staining were taken with a ×40 objective, and the staining quantitated as described in the “Methods” section. Representative images of Fluoro Jade C staining were taken with a ×20 objective, while cell counts were performed on three representative ×40 fields per mouse. *Error bars* = SEM, *p < 0.05, ****p < 0.0001

The ability of IgG and various leukocytes to effectively target the CNS is indicative of BBB pathology in MRL/lpr mice [8, 30]. In the present study, BBB disruption was assessed by extravasation of fibronectin, a serum protein not widely distributed in the CNS [31]. Fibronectin leakage was observed across the BBB in over half of MRL/+ → MRL/lpr and MRL/lpr → MRL/lpr chimeras, while no evidence of leakage was found in MRL/+ → MRL/+ mice (F_4,27_ = 4.25; *p* = 0.01, Fig. 3a). Thus, an abnormally permeable BBB occurred independently of systemic inflammation.

#### MRL/+ → MRL/lpr mice exhibit increased cortical neurodegeneration

FJC staining is an effective means of visualizing neurodegeneration from various etiologies [32]. While differences in hippocampal neurodegeneration were previously observed between 16-week-old MRL/lpr and MRL/+ mice [27], we found no such differences, perhaps since the mice studied here were significantly older (27 weeks old). Nevertheless, increased FJC cortical staining in MRL/lpr → MRL/lpr and MRL/+ → MRL/lpr mice, as compared to MRL/+ → MRL/+ mice, was present (F_2,27_ = 4.25; *p* < 0.0001, Fig. 3b).

#### RANTES is similarly overexpressed in the brains of MRL/+ → MRL/lpr and MRL/lpr → MRL/lpr mice

**Fig. 4:**
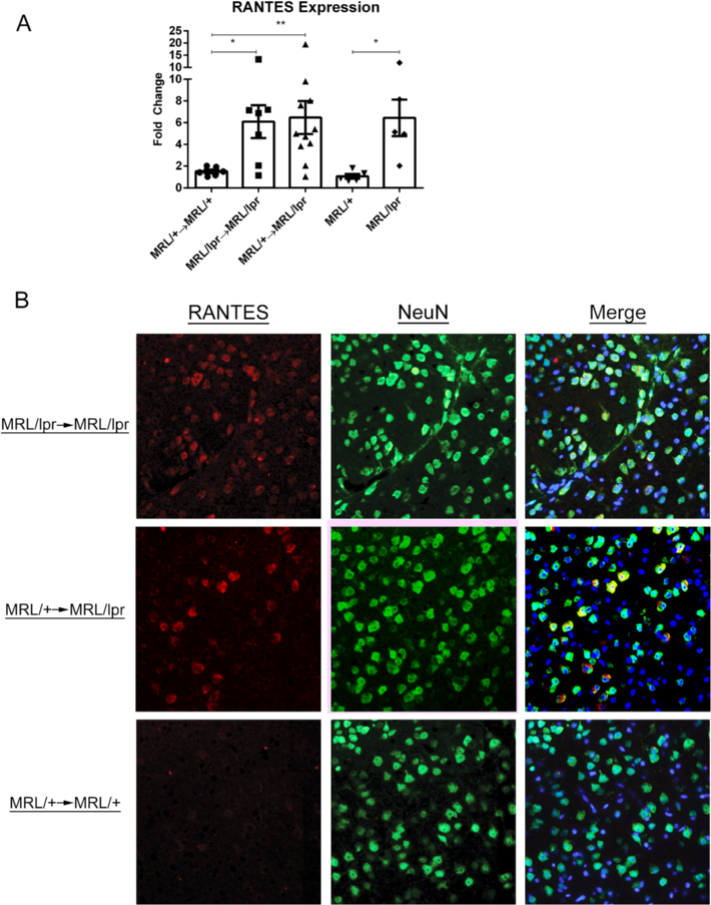
Increased neuronal RANTES in MRL + →MRL/lpr mice. Real time quantitative PCR revealed significantly increased brain RANTES expression in MRL/lpr background mice (**a**), which by immunofluorescence was found to be produced mostly by neurons (**b**). Representative images taken with a ×40 objective are shown. *Error bars* = SEM, *p < 0.05, **p < 0.01

RANTES-mediated chemotaxis and inflammation is important in the development of multiple manifestations of SLE, including cutaneous [33], renal [34], and neuropsychiatric presentations [18, 35, 36]. Significant differences were found in RANTES gene expression in MRL/+ → MRL/lpr, MRL/lpr → MRL/lpr, and MRL/lpr mice, relative to both MRL/+ → MRL/+ and MRL/+ mice (H = 8.606; *p* < 0.02, Fig. 4a). Immunofluorescent staining confirmed increased RANTES expression, which was predominately localized to layer V cortical neurons in MRL/+ → MRL/lpr and MRL/lpr → MRL/lpr mice (Fig. 4b). Additionally, there was non-neuronal cellular staining within and around vasculature of both the cortex and hippocampus and a punctate, granular staining pattern in the neuropil (data not shown).

#### Choroid plexus infiltrates persist in MRL/+ → MRL/lpr chimeras despite normalization of peripheral autoimmunity

**Fig. 5:**
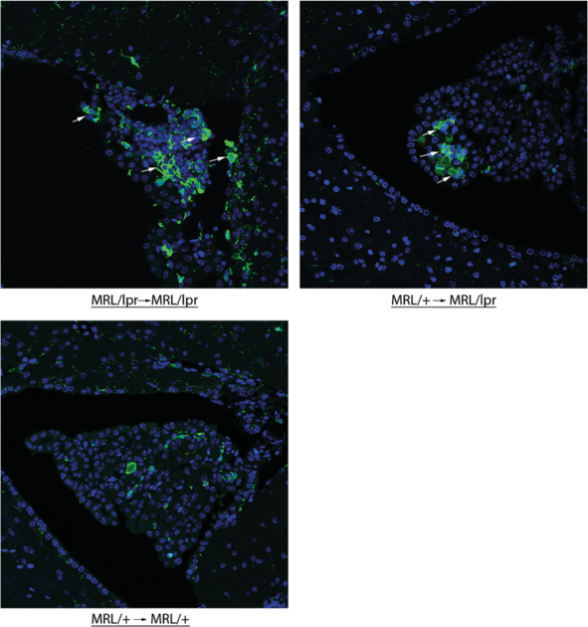
Monocytic infiltrates in the choroid plexus of MRL + →MRL/lpr mice. IBA-1 staining revealed a mixed cellular infiltrate including IBA-1+ cells in the choroid plexus of MRL/lpr → MRL/lpr and MRL/+ → MRL/lpr mice but not in MLR/+ → MRL/+ mice. Representative images taken with a ×40 objective are shown

Previous studies have demonstrated increased F4/80 expression as a marker of activated microglia in MRL/lpr mice, predominately in the hippocampus, although cortical nests of activated microglia were found as well [27]. In the present study we found no significant differences in hippocampal IBA-1 staining between chimeric mice (F_2,22_ = 2.262; ns) although significant increases were found in unmanipulated MRL/lpr compared to MRL/+ mice (*p* = 0.01; data not shown). In nearly all MRL/+ → MRL/lpr and MRL/lpr → MRL/lpr mice, however, we did find an increase in cellular infiltration elsewhere in the brain, while no such infiltrates were found in MRL/+ → MRL/+ or MRL/+ mice. These infiltrates, largely consisting of IBA-1+ cells, were found in the choroid plexus of the dorsal fourth ventricle, as well as proximal to the hippocampus (Fig. 5).

#### Brain IgG deposition is not increased in MRL/+ → MRL/lpr mice

**Fig. 6:**
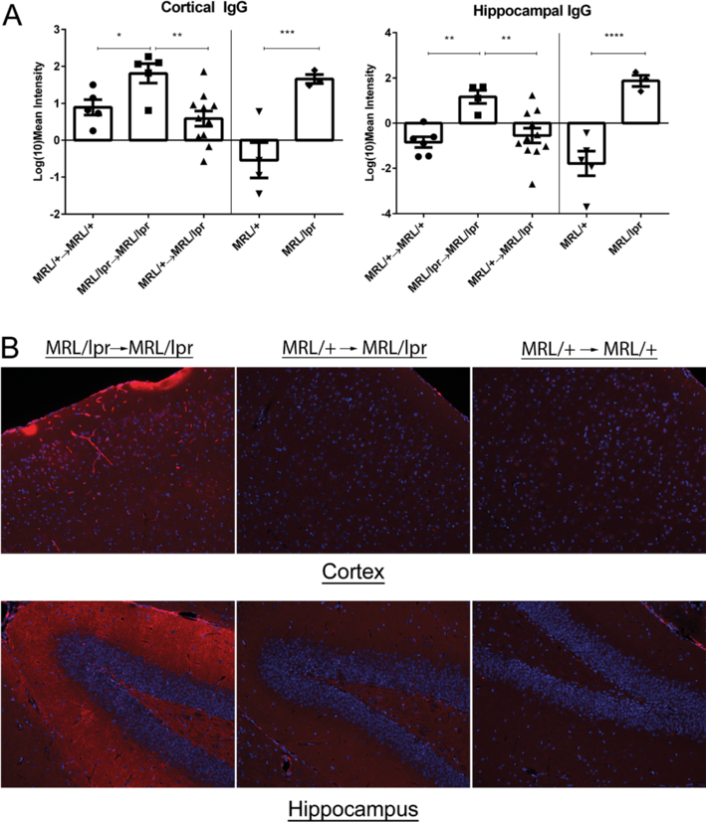
IgG deposition is reduced in the cortex and hippocampus of MRL/+ → MRL/lpr chimeric mice. Immunofluorescent staining, quantitated in (**a**) absolute values of mean intensity, demonstrated significantly increased tissue penetration and deposition of IgG in MRL/lpr → MRL/lpr mice as compared to MLR/+ → MRL/+ and MRL/+ → MRL/lpr mice in both the cortex and the hippocampus. Representative images taken with a ×20 objective are shown in (**b**). *Error bars* = SEM, *p < 0.05, **p < 0.01, ****p < 0.0001

Local immune complex formation by autoantibodies binding to neurally expressed antigens is thought to be involved in the pathogenesis of NPSLE. Histological evaluation of IgG deposition in both cortical (F_4,23_ = 8.9; *p* = 0.002) and hippocampal (F_4,24_ = 11; *p* < 0.0001) brain regions revealed that both MRL/lpr → MRL/lpr and MRL/lpr mice had significantly higher IgG tissue deposition than MRL/+ → MRL/+, MRL/+ → MRL/lpr, and MRL/+ mice (Fig. 6).

Taken together, our results show that MRL/+ → MRL/lpr chimeric mice replicate the key features of NPSLE found in the parent MRL/lpr strain, including neurobehavioral deficits, a breached BBB, neurodegeneration, and inflammatory infiltrates in the choroid plexus, despite normalization of key autoimmune features in the periphery, as well as decreased IgG deposition within CNS tissue.

## Discussion

Attempts to identify associations between humoral inflammatory effectors and NPSLE have not been able to conclusively demonstrate causality to date [37, 38]. The study presented herein utilized BMT to separate innate CNS from peripheral hematopoietic contributors to the development of NPSLE. Through transplantation of healthy control MRL/+ BM to lupus-prone MRL/lpr mice, we significantly attenuated the development of systemic autoimmunity and renal disease. Nevertheless, MRL/+ → MRL/lpr chimeras displayed a neuropsychiatric profile consistent with MRL/lpr → MRL/lpr chimeric and unmanipulated MRL/lpr mice, in the absence of any motor abnormalities and with a preserved exploratory drive. We further found evidence of BBB disruption, neurodegeneration, and RANTES upregulation in both MRL/+ → MRL/lpr and MRL/lpr → MRL/lpr chimeras. Notably, there were no behavioral differences between syngeneic chimeras and unmanipulated mice strains, indicating that BMT did not itself significantly impact neuropsychiatric manifestations.

We previously have shown that neuropsychiatric manifestations in MRL/lpr mice occur early [10] and may, therefore, theoretically have been established by the time the BMT procedure was performed. While we do not have incontrovertible evidence to disprove this hypothesis, given the persistent NPSLE phenotype absent concomitant systemic autoimmunity, we nevertheless report here for the first time that NPSLE may develop (or persist) without significant contributions from a sustained autoantibody response. Thus, normalization of the systemic immune response did not affect the molecular pathways involved in neurobehavioral deficits in this strain. Although it is possible that the NPSLE phenotype in MRL/+ → MRL/lpr was contributed to by systemic cytokines present in the MRL/lpr background rather than brain-intrinsic factors, the normalized autoantibody titers and renal disease suggest that BMT controlled the systemic inflammatory response as well. Unfortunately, BMT could not be done before 8 weeks of age for technical reasons, and repeated behavioral testing would be non-informative since initial testing affects subsequent performance.

Autoreactive antibodies binding to the endothelium, kidneys, and skin are among the major effectors of the vascular, renal, and cutaneous manifestations of SLE, respectively [39–42]. Consequently, many researchers have searched for autoantibodies binding to neural antigenic determinants in NPSLE. Anti-NMDAR antibodies have been found in SLE patients [28] and can induce NPSLE-like manifestations upon transfer to mice when coupled with disruption of the BBB [43]. Anti-ribosomal-P antibodies have been linked with human and experimental NPSLE as well [44]. There is indeed no doubt from these and other studies that antibodies given intrathecally or systemically can replicate some of the features of lupus-associated neuropsychiatric disease. Moreover, the single strain studied here does not necessarily represent the full complexity of experimental neuropsychiatric lupus. Nevertheless, the MRL/lpr is a spontaneous lupus strain and a pivotal model in the investigation of lupus-associated memory abnormalities and behavioral deficits. Since we found that MRL/+ → MRL/lpr mice did not have elevated serum autoantibody titers while still exhibiting robust neuropsychiatric disease, it therefore appears that a sustained serum autoantibody response is not strictly required for the initiation or maintenance of a spontaneous NPSLE phenotype. The modest increases in autoantibody levels seen in MRL/+ → MRL/lpr chimeric mice were not likely neuropathic, since renal involvement, a lupus complication closely linked to circulating anti-dsDNA antibodies, was absent. Furthermore, correlation analysis between behavioral outcomes and systemic disease manifestations revealed no significant relationship (data not shown). We further evaluated whether anti-NMDAR antibodies were elevated in chimeric MRL/+ → MRL/lpr mice and found that titers were not significantly different from those of MRL/+ → MRL/+ chimeric mice (data not shown). Additionally, when evaluating IgG within the CNS parenchyma, we found that MRL/+ → MRL/lpr mice had minimal to no deposition, similar to MRL/+ → MRL/+ chimeric mice. Therefore, while autoantibodies may still affect disease progression and/or specific features in NPSLE, these were less critical in the behavioral phenotype found in chimeric MRL/+ → MRL/lpr mice. Furthermore, our model is entirely consistent with the variable seropositivity of NPSLE patients and the requisite BBB disruption or intrathecal administration used in autoantibody transfer models of NPSLE [45, 46]. Nevertheless, the absence of peripherally elevated autoantibodies does not wholly preclude intrathecal generation of antibodies binding to brain antigens. However, the low antibody and autoantibody titers found systemically in MRL/+ → MRL/lpr chimeras, as well as the low amounts of tissue bound IgG, make it distinctly unlikely that these mice were exposed to a significant increase in circulating brain reactive antibodies.

Depression-like behavioral manifestations of NPSLE in MRL/lpr mice were attenuated by treatment with cyclophosphamide, as measured by sucrose preference and forced swim tests [47, 48], which would suggest a dependence of CNS disease development on systemic autoimmunity. More recent work, however, has found that loss of sucrose preference in MRL/lpr mice is associated with peripheral taste receptor inflammation, which limits the utility of sucrose preference in characterizing the behavioral phenotype of MRL/lpr mice [49]. Furthermore, the effects of cyclophosphamide extend beyond its role as a cytotoxic alkylating agent, including anti-inflammatory and immunomodulatory activity within the CNS [50], which may contribute to the observed reduction in depression-like behavior.

Use of BM chimeras allowed us to take advantage of the fact that brain cells are relatively resistant to γ-irradiation and represent self-renewing cell populations with no significant peripheral contributors [51]. The inflammatory mechanisms uncovered would, therefore, be related to endogenous CNS effectors. RANTES, a potent T cell chemoattractant that has been previously found to be a significant component of renal, cutaneous, and neuropsychiatric lupus manifestations, was overexpressed in MRL/lpr host mice primarily by neurons, regardless of transplant condition. Interestingly, when stimulated by RANTES in vitro, neurons display modified expression of genes involved in synaptogenesis and neurite growth [52], suggesting that RANTES signaling may contribute to the cognitive manifestations found in MRL/lpr mice. This potential mechanism warrants further exploration in future studies.

MRL/lpr mice were previously reported to display increased hippocampal F4/80+ microglia staining [27]. When quantitating IBA-1 staining within the hippocampus, we found no difference between groups of chimeric mice. It is important to highlight that microglia are known to be a self-renewing cell population that is highly resistant to ionizing radiation, while peripherally derived macrophages are not [53, 54], suggesting that our findings are not a consequence of the BMT itself. We did, however, find increased IBA-1 staining in unmanipulated MRL/lpr mice (data not shown). Moreover, we identified infiltrating cells through the choroid plexus and para-hippocampal vasculature in MRL/+ → MRL/lpr, MRL/lpr → MRL/lpr, and unmanipulated MRL/lpr mice, many of which were IBA-1+. As MRL/+ → MRL/lpr mice lacked significant systemic disease and would therefore not be expected to have an abnormally hyperactive peripheral immune system, cellular infiltrates further point to CNS-driven inflammation and chemotaxis (possibly through RANTES) as essential to NPSLE development.

One potential limitation of the experimental design is the challenge in generating complete chimerism. This is particularly difficult in MRL/lpr mice, as they are systemically radiosensitive although hematopoietically radioresistant [55]. Nevertheless, the split-dose BMT method utilized herein has previously been shown to generate over 95 % engraftment [56]. More importantly, MRL/+ → MRL/lpr chimeras had the same systemic immune profile as MRL/+ → MRL/+ mice, indicating successful engraftment and repopulation by donor cells. Another possible limitation was the lack of head shielding during irradiation. Given that the vast majority of neurogenesis occurs within the hippocampus, the lack of head shielding, while increasing the degree of chimerism, may theoretically have obscured any differences in neurodegeneration in that region. Nevertheless, both MRL/+ → MRL/+ and MRL/lpr → MRL/lpr control chimeras were virtually indistinct behaviorally, systemically, and neuroimmunopathologically from their unmanipulated MRL/+ and MRL/lpr counterparts, respectively, further highlighting both that chimera generation was successful and that the lack of head shielding had no appreciable deleterious effects on study outcome.

A point of interest is the potential role of the hypothalamus–pituitary–adrenal (HPA) axis in development of the MRL/lpr NPSLE phenotype. Previous studies have shown increased HPA axis activity in this strain, evidenced by increased serum corticosterone levels as well as adrenal hyperplasia [57, 58]. Furthermore, treatment with immunosuppression normalized expression of neuroendocrine mediators in MRL/lpr mice, suggesting that neuroendocrine dysregulation is consequent to autoimmunity [59]. Given that systemic autoimmunity was highly attenuated in chimeric MRL/+ → MRL/lpr mice, the behavioral phenotype present in these mice is not likely attributable to neuroendocrine deficits.

An additional question is the potential role of CD4/CD8 double negative T cells (DN T cells), a cell type characteristically overrepresented in the unmanipulated MRL/lpr strain. In general, little is certain regarding the contribution of DN T cells to the pathogenesis of SLE in MRL/lpr mice, which may include both innate inflammatory as well as regulatory activity. However, given that DN T cells in this strain develop as a consequence of the *Fas* mutation [60] and that chimeric mice received *Fas* wild type bone marrow, it is highly unlikely that DN T cells played a major role in the pathogenesis of the neurobehavioral phenotype. Nevertheless, while outside the scope of the current study, whether double negative T cells are present within the CNS of unmanipulated and/or chimeric mice is an interesting question that can be addressed in the future.

Identification of appropriate unmanipulated control mice for this study was challenging. As the MRL/lpr → MRL/lpr chimeric mice underwent total body irradiation and disruption of their immune systems at 8 weeks of age, they experienced atypical longevity. Additionally, 21 days after BMT, donor leukocyte counts approach normal levels in peripheral blood [61], implying that immunological age of chimeric mice would be 11–12 weeks younger than their physiological age. In an attempt to compromise between “immunological” and physiological age, we chose to age-match unmanipulated controls at 8 weeks younger than chimeric mice, which would bias the control mice toward increased autoimmunity, thereby allowing for more confidence when comparing the immune activity between groups. In any case, it is noteworthy that the control chimeras (MRL/+/→MRL/+ and MRL/lpr → MRL/lpr) were phenotypically indistinct from unmanipulated control mice.

## Conclusions

Human NPSLE is challenging to treat, particularly since clinicians have incomplete information regarding the relative contributions of the several potential mechanisms involved. Acute disease exacerbations are often treated with high-dose corticosteroids, a problematic therapeutic option in NPSLE patients due to the frequent psychiatric and other unwanted side effects associated with this medication. While certain diffuse NPSLE presentations are treated empirically with mixed success, there are only few evidence-based treatment protocols [62–64]. There have long been questions as to whether NPSLE is a primary manifestation of SLE or a consequence of systemic disease. Neuropsychiatric signs can be the initial presentation of patients ultimately diagnosed with SLE and are similarly among the earliest abnormalities identified in young MRL/lpr mice [9, 10]. This chronology of NPSLE development, along with maintenance of the phenotype despite long term normalization of circulating autoantibody titers demonstrated here, collectively suggest that NPSLE can develop or persist along a pathway quite distinct from systemic disease manifestations. We acknowledge that systemic immunosuppressive treatment can be effective in human NPSLE and has an effect in murine models as well. It is nevertheless implicit in our results that therapies directed at putative pathogenic autoantibodies or systemic cytokines may be enhanced by additionally targeting CNS specific pathways, the nature of which remain to be discovered. Moreover, our results may have significance beyond understanding the pathogenesis of brain involvement in SLE, but may also provide important insight into understanding other autoimmune disorders with mixed brain/systemic presentations, such as Sjogren’s syndrome and HIV-associated neurocognitive disorders.
